# Determinants and Drivers of Infectious Disease Threat Events in Europe

**DOI:** 10.3201/eid2204.151073

**Published:** 2016-04

**Authors:** Jan C. Semenza, Elisabet Lindgren, Laszlo Balkanyi, Laura Espinosa, My S. Almqvist, Pasi Penttinen, Joacim Rocklöv

**Affiliations:** European Centre for Disease Prevention and Control, Stockholm, Sweden (J.C. Semenza, L. Balkanyi, L. Espinosa, P. Penttinen);; Stockholm University Stockholm Resilience Centre, Stockholm (E. Lindgren, M.S. Almqvist); Umeå University, Umeå, Sweden (J. Rocklöv)

**Keywords:** infectious diseases, drivers, determinants, globalization, environment, social, demographic, public health systems, travel, tourism, food and water, natural environment, global trade, climate change, antimicrobial resistance, Europe

## Abstract

Infectious disease threat events (IDTEs) are increasing in frequency worldwide. We analyzed underlying drivers of 116 IDTEs detected in Europe during 2008–2013 by epidemic intelligence at the European Centre of Disease Prevention and Control. Seventeen drivers were identified and categorized into 3 groups: globalization and environment, sociodemographic, and public health systems. A combination of >2 drivers was responsible for most IDTEs. The driver category globalization and environment contributed to 61% of individual IDTEs, and the top 5 individual drivers of all IDTEs were travel and tourism, food and water quality, natural environment, global trade, and climate. Hierarchical cluster analysis of all drivers identified travel and tourism as a distinctly separate driver. Monitoring and modeling such disease drivers can help anticipate future IDTEs and strengthen control measures. More important, intervening directly on these underlying drivers can diminish the likelihood of the occurrence of an IDTE and reduce the associated human and economic costs.

## Abstract 2

Globalization and environment, the most frequent underlying drivers, should be targeted for interventions to prevent such events.

The Middle East respiratory syndrome coronavirus (MERS-CoV) outbreak and the large Ebola outbreak in West Africa are striking examples of how emerging and reemerging infectious diseases can threaten international public health and strain governmental resources (*1*,*2*). Historically, novel pathogens have emerged and reemerged repeatedly in human populations and affected public health; similarly, pathogens that have been present in a population at low levels have increased rapidly in incidence or geographic range with equally grave consequences (*3*). The context of infectious disease emergence has changed over the centuries, but Europe has remained and even intensified as a hot spot for emerging infectious diseases over recent decades (*4*). Many of the fundamental and basic determinants of emerging infectious diseases have persisted over time, but dynamic global trends provide more opportunities for emerging infectious diseases to occur and expand swiftly (*5*,*6*).

A 2008 study, which was conducted by the European Centre for Disease Prevention and Control (ECDC) and based on expert consultation and literature review, projecting how the risk of emerging infectious diseases in Europe will be shaped in the future determined that drivers can be categorized into 3 main groups: globalization and environment, sociodemographic, and public health systems (Table 1) (*7*). Although the 3 groups are somewhat artificial and not entirely mutually exclusive, they can serve as a framework for the interpretation of infectious disease threat events (IDTEs) in Europe.

**Table 1:** Determinants and drivers of infectious disease threat events, Europe, 2008–2013

Drivers, by group	Examples*
Globalization and environment	
Climate	Temperature, humidity, wind, rainfall. Can have an effect on exposure pathways of foodborne and waterborne diseases or the distribution of vectorborne diseases.
Natural environment	Land cover, vegetation, water ways, oceans, coastlines, water resources, land use, habitats, biodiversity. Can shift the distribution range and influence abundance of vectors (e.g., rodents, mosquitoes, ticks) as well as of host and reservoir animals.
Human-made environment	Urbanization, built environment, infrastructure, industries, intensive agriculture. Can enable propagation and dissemination of pathogens.
Travel and tourism	Movement of populations by automobile, train, ship, airplane. Can enable the importation of vectors, pathogens and infected persons into Europe and their dispersion within Europe.
Migration	Immigrant, emigrant, asylum seeker, settler. Can be vulnerable to or contribute to spread of infectious diseases in origin country, in transit, or in destination country.
Global trade	Import and export of goods and services across international boundaries via ship, airplane, rail, truck. Can result in the exportation or importation (on purpose or involuntarily) of host animals, disease vectors, or pathogens.
Sociodemographic	
Demographic	Population composition with regards to age, income, education. Can be associated with greater health vulnerabilities.
Social inequality	Uneven distribution of resources in society, including income, wealth, rights, privileges, social power, education. Disadvantaged groups can suffer disproportionately from infectious diseases.
Vulnerable groups	Children, premature infants, pregnant women, elderly persons, men who have sex with men, immunocompromised persons. Vulnerability can increase exposure and susceptibility to infectious diseases or decrease access to care and recovery.
Prevention	Childhood vaccination programs, adherence to treatment regimes, appropriate prescription practices. Distrust in prevention efforts can undermine control efforts (e.g., childhood vaccination programs. Neglect of prevention when traveling
Lifestyle	High-risk behavior, such as intravenous drug use or unprotected sex with multiple partners. Can increase exposure and infection rates.
Occupational	Healthcare workers, veterinary and animal care personnel, butchers, farmers, cleaners. Lapses in infection control practices can put healthcare workers at risk.
Terrorism	Intentional release or dissemination of biologic agents. Intentional contamination of drinking water can result in community outbreaks.
Public health systems	
Healthcare system	European healthcare structure for the delivery of health services, including general practitioners, hospitals, clinics. Access to care, medicines, diagnostics, insurance coverage, for example, can affect health outcomes. Healthcare systems contribute to nosocomial infections.
Animal Health	Veterinary services, animal health and welfare measures, intensive livestock practices. High animal densities can promote infectious disease transmission. Infected animals close to human settlements can increase the risk for zoonotic epidemics.
Food and water quality	Agriculture, husbandry, farming, processing, handling, preparation and storage of food, man-made water systems (e.g., cooling towers, hot and cold water systems, spa pools, humidifiers), water treatment and distribution systems. Contamination of drinking and irrigation water sources and water distribution systems can result in both localized and community outbreaks. Contamination of foodstuff along the chain from farm to fork can result in multistate epidemics
Surveillance and reporting failure	Systematic ongoing collection, collation, analysis, and dissemination of infectious disease data. Lapses in surveillance can impede a rapid response to infectious disease outbreaks. In contrast, increased surveillance will contribute to increased awareness and thus result in increased reporting of cases
*Examples are purposely not exhaustive and should be considered illustrative.	

We conducted this study to identify, differentiate, and rank drivers of observed IDTEs in Europe detected through ECDC epidemic intelligence activities. By doing this, the disparate drivers that act on different dimensions and different scales can be disaggregated. A ranking of the relative importance of these drivers can help prioritize risk-based surveillance to anticipate disease emergence and spread (*8*,*9*). The effect of IDTEs on public health can be attenuated by strengthening the detection of and early response to the threats. However, more important, the likelihood of IDTEs originating in the first place can be reduced by intervening directly on their underlying drivers. Mitigation strategies to reduce the causes rather than the effects of IDTEs can be more cost effective (*10*).

## Event-Based Surveillance

Persons, services, goods, capital, and microbes are free to move across borders of the European Union (EU), which currently has 28 member states and an estimated population of 508.2 million. The ECDC is an EU agency with a mission to identify, assess, and communicate current and emerging threats to human health posed by infectious diseases. This charge is accomplished through epidemic intelligence, a process to detect, verify, analyze, assess, and investigate events that may represent a threat to public health. These activities are conducted by a team of >10 epidemiologists in the Emergency Operation Center at ECDC. The daily activity of epidemic intelligence at ECDC involves active or automated web searches from confidential and official sources (e.g., EWRS [Early Warning and Response System], ProMED [Program for Monitoring Emerging Diseases], MediSys [Medical Information System], and GPHIN [Global Public Health Intelligence Network]), as well as individual reports from the EU and European Economic Area (i.e., EU countries plus Iceland, Liechtenstein, and Norway) member states. The sources of epidemic intelligence information include several websites and a large number of webpages retrieved through specialized search engines. Additional information is gathered through direct contact with epidemiologists and health authorities in the EU and abroad.

Data collection for epidemic intelligence at ECDC was standardized in 2008; thus, we analyzed the epidemiologic characteristics of each IDTE in Europe from July 1, 2008, through December 31, 2013. For each IDTE the following data are routinely collected by ECDC: type of disease or pathogen, geographic location of source of infection, source of infection (e.g., contaminated bean sprouts), duration of the epidemic or of surveillance activities, number of countries affected by the event, number of cases, and number of deaths. IDTEs included in this study were restricted to outbreaks affecting >5 persons in the EU (excluding Croatia, which was not yet an EU member). Persons infected abroad and returning to the EU were included in our analysis. The IDTEs were sorted into 10 categories (Table 2).

**Table 2:** Infectious disease threat events detected in Europe, 2008–2013

Threat event category	Definition and examples*
Foodborne and waterborne	All types of diseases caused by the transmission of organisms through food or water (e.g., drinking water, recreational water): salmonellosis, hepatitis A, *Escherichia coli* infection, norovirus infection, shigellosis.
Vectorborne and rodentborne	All vectorborne and rodentborne diseases (epidemics or first autochthonous cases): West Nile fever, malaria, dengue fever, Hantavirus infection.
Other zoonoses	Diseases caused by transmission of organisms through contact with animals or animal discharges: Q fever, cowpox disease, psittacosis.
Vaccine preventable	Main vaccine-preventable diseases that are normally part of the public health system’s vaccination programs: measles, pertussis, mumps (boys), rubella (girls).
Multidrug resistance associated	Emerging multidrug-resistant infections of public health concern: carbapenemase-producing *Enterobacteriaceae*, *Klebsiella pneumoniae*.
Healthcare associated	Infections contracted while hospitalized or transmitted through healthcare practices: meningococcal meningitis.
Injection drug use associated	Infections caused by injection drug use: botulism, HIV, anthrax.
Sexually transmitted	Emerging sexually transmitted diseases and increases in incidence of serious complications: meningococcal infections.
Influenza	Seasonal influenza and other pandemic influenzas.
Airborne	Respiratory diseases acquired through transmission of pathogens through air (e.g., particles, droplets): for example, legionellosis. Includes respiratory infections that can be transmitted through air or other pathways, including infections transmitted through aerosols, fomites, or direct contact: Middle East respiratory syndrome coronavirus.

*Examples are purposely not exhaustive and should be considered illustrative.

Information about the underlying drivers of these IDTEs was extracted from several sources: the Communicable Disease Threats Report (a weekly bulletin generated by the epidemic intelligence team at ECDC), epidemiologic reports and communications, rapid risk assessments, threat assessments, mission reports, and associated peer-reviewed publications retrieved from PubMed. The IDTE drivers were organized into 3 categories: globalization and environment, sociodemographic, and public health systems (Table 1) (*7*). Expert assessment, performed by the authors, was used to evaluate the quality and validity of the information regarding the drivers. Discordant assessments were resolved by consensus.

Drivers were subjected to descriptive analyses (individually or in combinations), including frequency rates and ranking of the drivers, in relation to different types of IDTEs. Euclidian distances (based on how the IDTE types occurred with the driver category in the empirical data) were also calculated between each 1 driver pair (*11*). The calculation of the distance between 2 drivers was derived from the multidimensional driver space, based on whether the drivers were present (1) or absent (0) during the emergence of an IDTE. We then used a set of dissimilarities for the distances between drivers to perform a hierarchical cluster analysis (*11*). Dissimilarity distances between clusters were recomputed by the Lance–Williams updated formula, according to the average clustering method, by using the statistical computing program R and the algorithms in the R Stats Package (*11*). The driver terrorism was not included here because only 1 threat event was linked to the driver. Dissimilarity between clusters of IDTE drivers were described graphically by using tree diagrams (dendrograms) to visualize the similarity and dissimilarity of drivers in the occurrence of the IDTE. The clustering algorithm applied to binary data has been reported to perform well (*12*). The distance between clusters measure how similar, or dissimilar, different drivers are in their co-occurrences in outbreaks.

## Determinants and Drivers of IDTEs

**Figure 1:**
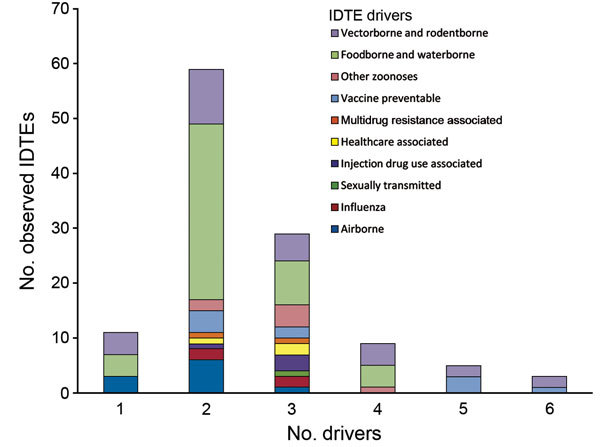
Number of observed infectious disease threat events (IDTEs) in relation to number of drivers for each IDTE group, Europe, 2008–2013.

**Figure 2:**
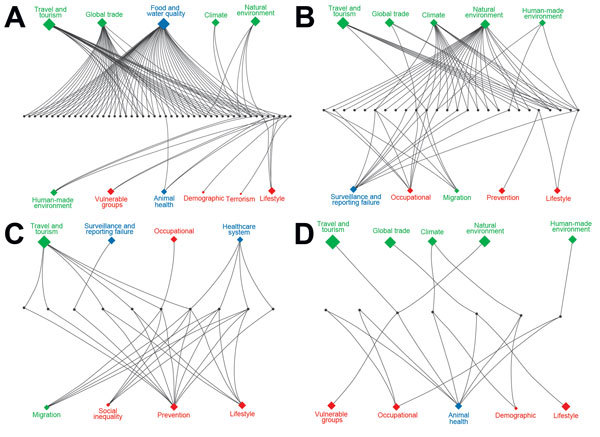
Infectious disease threat events (IDTEs), by contributing drivers, observed in Europe, 2008–2013. The 3 IDTE categories are represented by green (globalization and environment), red (sociodemographic), and blue (public health systems) symbols, the sizes of which are proportional to the overall frequency of the driver. A) Foodborne and waterborne IDTEs. B) Vectorborne and rodentborne IDTEs. C) Other zoonoses IDTEs. D) Vaccine preventable IDTEs.

 Of 274 IDTEs that occurred within the EU during July 2008–December 2013, a total of 116 met the study inclusion criteria. Foodborne and waterborne IDTEs were the most frequently occurring events (n = 48), followed by vectorborne and rodentborne IDTEs (n = 27), airborne IDTEs (n = 10) vaccine preventable IDTEs (n = 10), other zoonotic IDTEs (n = 7), injection drug use–associated IDTEs (n = 4), influenza IDTEs (n = 4), healthcare-associated IDTEs (n = 3), multidrug resistance–associated IDTEs (n = 2), and sexually transmitted IDTE (n = 1). The driver category that was by far the most frequently involved in single IDTEs was globalization and environment (61%), followed by the public health system failure (21%) and sociodemographic (18%) groups. The individual driver travel and tourism was linked to 9 of the 10 IDTE categories, and the vulnerable groups and lifestyle driver categories were linked to 7 and 6 IDTE categories, respectively. Most IDTEs had a combination of drivers: 51% had 2 drivers, and 25% had 3 drivers (Figure 1). Foodborne and waterborne diseases and vectorborne diseases were the most commonly occurring drivers in combinations of 2 and 3 drivers. The most common driver combinations were travel and tourism in combination with food and water quality and global trade in combination with food quality, both of which caused foodborne and waterborne diseases (Figure 2).

## Foodborne and Waterborne IDTEs

The foodborne and waterborne category included all types of diseases transmitted through food or water (Table 1) and was responsible for the most IDTEs. The global burden of foodborne diseases is considered to be substantial, although no current estimates exist (*13*). The most common cause of the observed foodborne and waterborne outbreaks in Europe was norovirus, followed by hepatitis A and *Escherichia coli* (hemolytic uremic syndrome and Shiga-like toxin–producing *E. coli* infections) (*14*,*15*). The strongest driver in this IDTE group was food and water quality, implicating the food industry and water treatment infrastructure, often in combination with the travel and tourism or global trade drivers (Figure 2, panel A). An example was the norovirus epidemic that affected >11,000 schoolchildren in 6 countries; the origin of the epidemic was traced to contaminated frozen strawberries (*16*).

## Vectorborne and Rodentborne IDTEs

Nearly half of the 27 IDTEs within the vectorborne and rodentborne IDTE category were caused by West Nile virus (WNV) infections. Four of these IDTEs consisted of the first autochthonous WNV cases in 4 different European countries and 1 large outbreak in southeastern Europe with >260 cases (*16*). WNV infection and malaria are notifiable diseases in the EU and, thus, subject to indicator surveillance; however, special threat events are picked up by event-based surveillance as well. The natural environment driver was present in all WNV infection events; in half of those events, it was present with the climate driver, and in 6 events, it was present with the surveillance and reporting failure driver (Figure 2, panel B). This finding is consistent with other findings that show environmental and climatic determinants play contributing roles in WNV infection outbreaks (*17*). Of 7 malaria threat events, 5 included autochthonous cases (in Spain, Greece, and Belgium). Our data also included the large dengue outbreak in Madeira, Portugal, with >2,000 cases (*18*) driven by climate, natural environment, and travel and tourism (Figure 2, panel B). A large outbreak of hantavirus infections in Germany in 2010 was attributed to bank vole (*Clethrionomys glareolus*) populations, which had increased substantially due to excessive seed production the previous year (*19*); human behavior (e.g., outdoor activities in summer); dust contaminated with rodent excreta following dry and warm weather; and heightened awareness, with better diagnosis and reporting.

## Other Zoonoses IDTEs

Q fever, psittacosis, and diseases caused by cowpox virus and *E. coli* (with an unusual transmission pathway, e.g., contaminated farm soil and petting of contaminated animals) were included in the other zoonoses IDTE category. Outbreaks often occurred among farm and animal workers. However, the 2007–2009 Q fever outbreak in the Netherlands affected >3,000 persons in densely populated areas (demographic driver) situated in close proximity to commercial dairy goat farms (climate and animal health drivers) (Figure 2, panel C) (*20*). Contaminated dust particles from ruminant farms probably caused airborne transmission of *Coxiella burnetii*, the causative agent of Q fever. Vaccination, hygiene measures, and culling of pregnant animals on affected farms eventually ended the outbreak (*21*). Due to persistence of *C. burnetii* in the environment, continued surveillance for Q fever is warranted.

In autumn 2009, an outbreak of 93 cases of *E. coli* O157 (verotoxin-producing *E. coli*) infection in southern England was related to environmental and animal exposure on a petting farm visited by families and children (tourism, vulnerable groups, animal health, and natural environment drivers) (Figure 2, panel C) (*22*). Horizontal integration of the human, animal, and environmental health sectors, according to the One Health approach, can tackle some of these public health predicaments (*23*).

## Vaccine-Preventable IDTEs

**Figure 3:**
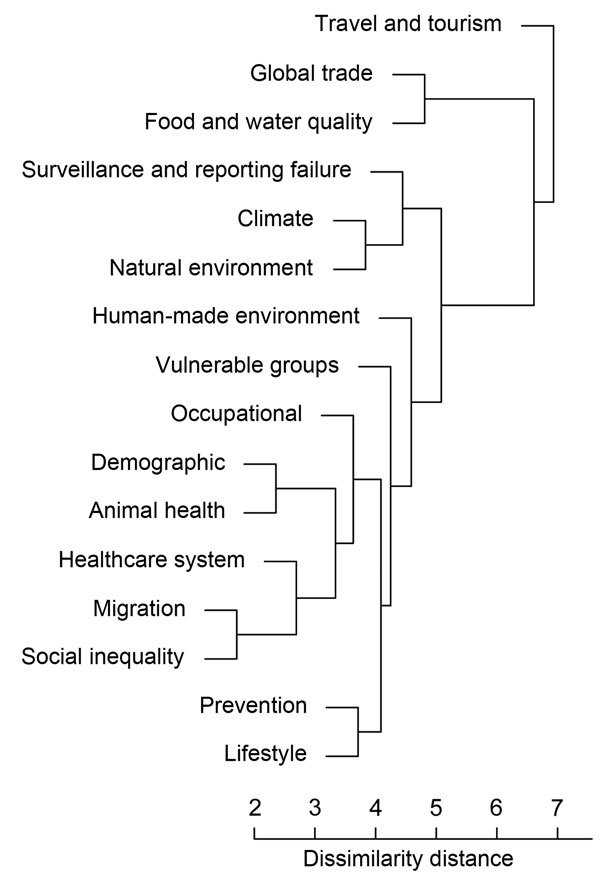
Cluster dendrogram from hierarchical cluster analysis of drivers contributing to observed infectious disease threat events (IDTEs), Europe, 2008–2013. Individual segments (leaves) on the lower part of the tree are more related to each other, as indicated by distances between the branches. Drivers below travel and tourism also occurred less often as underlying drivers of IDTEs and tended to be more contextual in nature. Scale bar indicates dissimilarity distance for drivers, as measured by frequency of pairwise co-occurrence in clusters. Similar drivers (e.g., that co-occurred in outbreaks) are at a close distance, and those that were more independent of other drivers show higher dissimilarity.

Ten IDTEs, including measles, mumps, rubella, and pertussis outbreaks, were reported for the vaccine-preventable IDTE category. A measles outbreak in Bulgaria in 2009–2010, which affected predominantly migrant and hard-to-reach Roma populations, resulted in >24,000 cases and 24 deaths in 1 year (*24*). The drivers responsible for this outbreak were a combination of prevention, lifestyle, migration, social inequality, and healthcare system (Figure 2, panel D). Measles is still endemic in many European countries because of low vaccination coverage among migrants and hard-to-reach populations and vaccine hesitancy. Specific vaccination strategies are often necessary for these populations to protect children and adults from infectious diseases, prevent spread of infection due to crowded living conditions, and ensure continuity of childhood immunization schedules. Failure to vaccinate susceptible populations diminishes herd immunity and may trigger outbreaks (*24*). There was no evidence of an increased risk of infectious disease transmission in the host population in Europe during the 2015 influx of migrants or asylum seekers; however, ECDC advocated for the implementation of basic public health measures, health assessments, and vaccination to address the health needs of migrants (*25*). This strategy is supported by the findings from this analysis, in which migration was a comparatively infrequent driver of IDTEs, relative to travel and tourism (Figure 3).

## Multidrug Resistance– and Healthcare-Associated IDTEs

Multidrug-resistant tuberculosis cases were identified as single case events and therefore did not meet our inclusion criteria in this study. The events in the multidrug resistance–associated IDTE group consisted of a nosocomial outbreak of multidrug-resistant carbapenemase-producing *Klebsiella pneumoniae* infection in 2 hospitals in Ireland and 69 infections with New Delhi metallo-β-lactamase-1 carbapenemase-producing *Enterobacteriaceae* in persons in the United Kingdom with a travel history to India or Pakistan (*26*).

The relatively few healthcare-associated IDTEs picked up by ECDC’s epidemic intelligence represent only a fraction of the expected number in Europe. Seven deaths occurred among 49 detected cases; 6 deaths were in newborns who had been infected while in hospitals (healthcare system, vulnerable groups, human-made environment drivers). The number of events and deaths for other event categories was far below the actual number expected for Europe. An ECDC point-prevalence survey of healthcare-associated infections and antimicrobial use in long-term care facilities in Europe showed that ≈4.1 million patients contract a healthcare-associated infection in the EU each year, and ≈37,000 deaths occur annually as a direct consequence of these infections (*27*). However, these types of infections were not captured by epidemic intelligence due to reporting disincentives (e.g., legal and financial).

## Injecting Drug Use–Associated IDTE

Reported infections caused by injection drug use were due to botulism, HIV, and anthrax and caused 69 illnesses and 8 deaths (*28*,*29*). As an example, a contaminated batch of heroin (global trade driver) caused 31 anthrax infections among heroin users in Scotland (vulnerable groups, lifestyle, sociodemographic drivers) (*30*).

## Sexually Transmitted IDTE

Only 1 sexually transmitted IDTE was identified by epidemic intelligence. The event was reported from 3 countries and consisted of invasive meningococcal disease among men who have sex with men; the men had been infected while traveling or through contacts from abroad. Of note, however, many sexually transmitted infections tend to be silent and reach endemic levels that are not captured by epidemic intelligence.

## Influenza IDTEs

Rather than registering as recurrent IDTEs, influenza precipitated several influenza outbreaks that were recorded as Public Health Events at ECDC; examples are the avian influenza A(H5N1) outbreaks and the influenza A(H1N1)pdm09 pandemic. Thus, the number of IDTEs attributed to influenza is underestimated. The drivers for the influenza IDTE group of both seasonal and pandemic influenza were travel and tourism, vulnerable groups, social and demographic, and surveillance and reporting.

## Airborne IDTEs

Ten IDTEs were reported for the airborne IDTE category. Most events were due to legionellosis, but the IDTEs also encompassed the emergence of MERS-CoV infections in 2012–2013; a total of 9 MERS cases were reported from the EU (*2*). Our analysis identified human-made environments, in particular contaminated cooling towers or spa pools, to be the overarching driver of *Legionella* infection events (*31*). Proper maintenance of the physical infrastructure can prevent these IDTEs. Two drivers, travel and tourism and healthcare system, were identified for the emergence and spread of MERS-CoV into Europe (*2*).

## Driver Ranking

An overall frequency ranking of all events ranked the individual contribution of the top 5 drivers in the following order: travel and tourism, food and water quality, natural environment, global trade, and climate. The hierarchical cluster analysis revealed travel and tourism to be separate from all the other drivers; thus, this driver can be considered distinct, indicating that the distribution of IDTEs within travel and tourism is significantly different from the distribution in the remaining clusters (Figure 3). The hierarchical cluster analysis revealed several similarly clustered segments, such as climate and natural environment and migration and social inequality, indicating that these drivers are more related to each other than to the other drivers.

## Limitations

Although sociodemographic and public health system drivers were less frequent in our analysis of IDTEs, they are nevertheless key contributors to the disease burden from infectious diseases in Europe (*32*). They may also be more directly amenable to interventions. However, epidemic intelligence detects IDTEs, not endemic infectious diseases, to which these drivers contribute substantially. Epidemic intelligence is heavily influenced by media coverage, geographic focus, length of the epidemic intelligence monitoring cycle, diagnostic procedures, and sensitivity of surveillance systems, among many other factors. The captured events are then filtered and verified before they are assessed and investigated. One event during the study period was categorized as bioterrorism, because sabotage was suspected due to coliform contamination of drinking water tanks at a hotel. If need be, a Public Health Event is declared to initiate control measures.

However, the ECDC screening of IDTEs is not designed to capture infectious diseases that do not reach outbreak levels or are not picked up by event monitoring (e.g., healthcare-associated or sexually transmitted infections). Therefore, our analysis pertains only to the drivers of IDTEs rather than endemic infectious diseases that are not recorded by epidemic intelligence. Long-term monitoring of the incidence, prevalence, or both of notifiable diseases is performed on a national level and reported through a different reporting system, the European Surveillance System at ECDC (*33*).

## Discussion

We found globalization and environment to be the most noteworthy driver category for IDTEs in Europe. More specifically, travel and tourism, food and water quality, natural environment, global trade, and climate were the top 5 drivers of all IDTEs identified through epidemic intelligence at ECDC. Among these, travel and tourism proved to be significantly distinct in the hierarchical cluster analysis and cluster dendrogram (Figure 3). In this analysis of epidemic intelligence data, travel and tourism was not only the most distinct but also the most recurrent driver implicated in the emergence of IDTEs. The volume of international travelers on commercial flights with a final destination in Europe has increased steadily over the years; >103 million travelers entered Europe in 2010 alone, and this number will probably continue to grow (*34*). International travel from areas with epidemic and endemic diseases has resulted in continuous importation of infected persons into Europe who can, for example, trigger outbreaks of airborne diseases. Similarly, pathogen introduction into competent vector populations can result in local transmission and threaten the safety of the blood supply (*35*). Restricting international travel in a globalized world to reduce the likelihood of IDTEs is both unrealistic and undesirable; however, monitoring and modeling air traffic patterns for pathogen importation risk can potentially accelerate early case detection and rapid response and effective control of IDTEs (*36*).

Food and water quality was the second most frequent driver of IDTEs in Europe. Suboptimal food safety systems, even if they are distant to the outbreak, become an international public health issue in an interconnected world in which food and humans move freely (Figure 2) (*37*). The occurrence of an IDTE can potentially be mitigated by addressing this driver. Fostering multisectorial collaboration between the food industry, public health, and environmental agencies can prevent IDTEs. High-density agricultural practices need to be subjected to stringent farm biosecurity and sanitary practices to prevent multinational outbreaks (*23*). Upgrading water treatment and distribution systems can prevent communitywide outbreaks (*38*).

Changes in the natural environment are increasing on a nonlinear scale with habitat destruction and loss of ecosystem services (http://www.esa.org). Monitoring and modeling environmental precursors of IDTEs can help to anticipate, or even forecast, an upsurge of IDTEs (*39*). The utility of such predictive models has been documented on several occasions: environmental drivers of IDTE with prediction tools have been made available by ECDC through the E3 (European Environment and Epidemiology) Geoportal (https://e3geoportal.ecdc.europa.eu/SitePages/Home.aspx) (*40*).

In summary, we have taken a systematic approach to categorize and rank the underlying drivers of observed IDTEs in Europe to help anticipate, respond to, and recover from probable, imminent, or current impacts of these events. Drivers of IDTEs can arise as epidemic precursors of IDTEs. Monitoring and modeling these drivers can serve as early warning systems of IDTEs and accelerate responses (*39*,*40*). However, it is desirable to proactively prevent possible public health emergencies rather than respond to IDTE after they have occurred. Thus, the most cost-effective strategy would be to directly tackle the underlying drivers of an IDTE rather than deal with the actual IDTE after the fact (*10*). Intervening directly on drivers may prevent the occurrence of IDTEs and reduce the human and economic cost associated with IDTEs.
